# Comparison of the effects of nitrogen-, sulfur- and combined nitrogen- and sulfur-deprivations on cell growth, lipid bodies and gene expressions in *Chlamydomonas reinhardtii* cc5373-sta6

**DOI:** 10.1186/s12896-023-00808-3

**Published:** 2023-09-08

**Authors:** David I. Gonzalez, Ruby A. Ynalvez

**Affiliations:** 1 Department of Biological Science, Vanderbilt University, 465 21st Ave S, Nashville, TN 37240 USA; 2 Department of Biology and Chemistry, Texas A&M International University, 5201 University Blvd, Laredo, TX 78041 USA

**Keywords:** *Chlamydomonas reinhardtii*, Biofuel, Nitrogen and sulfur deprivation, Triacylglycerol, Lipid accumulation

## Abstract

**Background:**

Biofuel research that aims to optimize growth conditions in microalgae is critically important. *Chlamydomonas reinhardtii* is a green microalga that offers advantages for biofuel production research. This study compares the effects of nitrogen-, sulfur-, and nitrogen and sulfur- deprivations on the *C. reinhardtii* starchless mutant cc5373-sta6. Specifically, it compares growth, lipid body accumulation, and expression levels of acetyl-CoA carboxylase (ACC) and phosphoenolpyruvate carboxylase (PEPC).

**Results:**

Among nutrient-deprived cells, TAP-S cells showed significantly higher total chlorophyll, cell density, and protein content at day 6 (p < 0.05). Confocal analysis showed a significantly higher number of lipid bodies in cells subjected to nutrient deprivation than in the control over the course of six days; N deprivation for six days significantly increased the size of lipid bodies (p < 0.01). In comparison with the control, significantly higher ACC expression was observed after 8 and 24 h of NS deprivation and only after 24 h with N deprivation. On the other hand, ACC and PEPC expression at 8 and 24 h of S deprivation was not significantly different from that in the control. A significantly lower PEPC expression was observed after 8 h of N and NS deprivation (p < 0.01), but a significantly higher PEPC expression was observed after 24 h (p < 0.01).

**Conclusions:**

Based on our findings, it would be optimum to cultivate cc5373-sta6 cells in nutrient deprived conditions (-N, -S or –NS) for four days; whereby there is cell growth, and both a high number of lipid bodies and a larger size of lipid bodies produced.

## Background

Global population growth is accompanied by a dramatic increase in energy demand; thus, it is inevitable for the availability of fossil fuels to decrease, resulting in rising energy prices worldwide [1–3]. In addition, the burning of fossil fuels contributes greatly to environmental pollution and climate change, and is a major public health concern [4–6]. There is a need to find alternative sources of renewable energy that will not compromise the environment and human health. Biofuel research that aims to optimize growth conditions in microalgae is critically important. The use of microalgae in biodiesel production has received considerable attention as a great option as a renewable energy source [7–9]. For example, the use of microalgae has many advantages over land plants; they are easier to grow, have a higher lipid productivity, and can be grown in unfavorable conditions [10–12]. However, there are limitations at the technological level, such as cultivation methods, lipid extraction, and cost of production. These have made the process of biofuel production from microalgae not yet economically viable for industrial production [9, 13, 14].

*Chlamydomonas reinhardtii* is a green microalga that offers advantages for biofuel production research. The advantages include rapid growth, short generation time, strong adaptability and easy cultivation [15, 16]. In addition, *C. reinhardtii* has been extensively genetically characterized, i.e., genetic manipulations enhanced algae triacylglycerol (TAG) biosynthesis [10, 11, 17–19]. Interestingly, *C. reinhardtii* has the ability to synthesize and accumulate significant quantities of lipids in the form of triacylglycerols (TAGs) under specific stress conditions, i.e., light intensity alterations, nutrient deprivation, and salinity stress [16–18, 20–23].

Studies have reported that nutrient deprivation in *C. reinhardtii* after four days results in a significant accumulation of TAGs [17, 18]. Specifically, nitrogen (N) starvation elicits the differentiation of algal cells into gametes, an accumulation of carbohydrates and an increase in lipid content [18, 24, 25]. On the other hand, sulfur (S) deprivation causes inactivation of photosynthetic activity, an increase in H_2_ production, and an increase in lipid content [17, 26]. Although there have been many studies on N deprivation as well as S deprivation, to our knowledge, no studies have reported how the combination of N and S deprivation affects cell growth, chlorophyll content and lipid production in *C. reinhardtii*. We hypothesize that the removal of both N and S in Tris-acetate-phosphate (TAP) growth media will further increase lipid biosynthesis in the starchless mutant *C. reinhardtii* cc5373-sta6 when compared to the removal of either N only or S only from the growth media.

When subjecting *C. reinhardtii* to stress, it is important to consider algal cell growth and the energy storage compounds that accumulate in *C. reinhardtii*. It is also known that alterations made to cellular metabolic pathways via genetic modifications in microalgae can either enhance or reduce specific metabolite production [10]. Mutants of *C. reinhardtii*, such as the starchless mutant cc5373-sta6, have higher lipid body output linked to increased carbon allocation towards lipid metabolism [10, 27, 28]. Under nutrient deprivation, the cc5373-sta6 strain has been shown to increase TAG content by 10-fold compared to the wild type [10, 29]. Although nutrient deprivation and lipid production have been studied in this strain, there is a need to further characterize and optimize its cultivation conditions to potentially address the limitations in the use of microalgae in biofuel production. The results of this study can provide further knowledge on cc5373-sta6’s potential as an alternative renewable source of energy for biofuel production.

The purpose of this study was to compare the effects of nitrogen (N), sulfur (S) and the combination of nitrogen and sulfur (NS) deprivation on cc5373-sta6 via chemical, microscopic and genetic analysis to establish optimal conditions for lipid body (TAG) biosynthesis in *C. reinhardtii*. The objectives of this study were to determine the effects of N-, S- and NS-deprivations on cc5373-sta6’s (1) growth by conducting chemical analysis (cell density, biomass accumulation, chlorophyll content and protein content), (2) lipid body accumulation (TAG) via confocal microscopy imaging analysis and (3) transcriptional expression levels of lipid metabolic enzymes, namely, acetyl-CoA carboxylase (ACC) and phosphoenolpyruvate carboxylase (PEPC), via real-time polymerase chain reaction (RT-PCR). This study will contribute to the scientific knowledge on (1) cc5373-sta6’s potential as a renewable source of energy and (2) the commercialization of microalgae by improving triacylglycerol (TAG) production in microalgae. Thus, the results of this study will be a relevant addition to the knowledge on microalgae lipid biosynthesis.

## Results and discussion

### Effects of N-, S- and NS-deprivations on cell growth, chlorophyll and protein content

#### Effects on cell growth

**Fig. 1:**
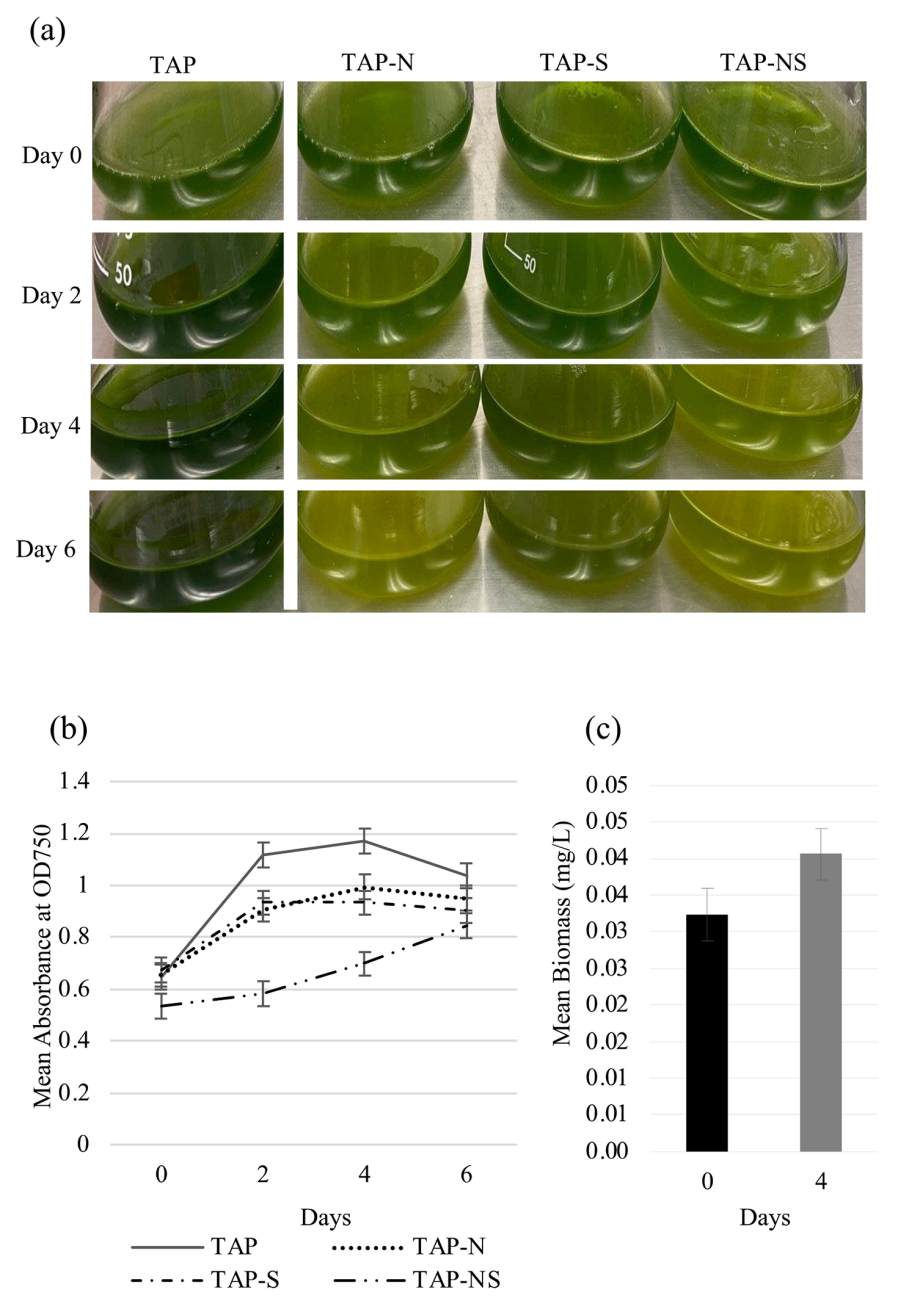
Growth analysis of cc5373-sta6 under conditions of nutrient deprivation (-N, -S and –NS) (**a**) cc5373-sta6 cells under different nutrient conditions and growth was monitored from day 0 to 6 (**b**) cell density of strain cc5373-sta6 was determined by measuring the optical density at OD_750_ every 48 h for six days after nutrient deprivation (**c**) biomass of strain cc5373-sta6 was determined after four days of nutrient deprivation; results from each nutrient deprivation condition were pooled since there was no significant difference observed among -N, -S and –NS. Bars in (**b**) and (**c**) represent 95% confidence intervals

To determine and compare the growth characteristics of cc5373-sta6 under nutrient deprivation (-N, -S and –NS), cell density measurements and biomass determinations were conducted. Cell density is typically measured at optical density (OD) of 750 nm in *C. reinhardtii* [30, 31]. This wavelength is out of the absorbance range of algal pigments [32]. Figure 1a shows qualitative differences in cell growth among cc5373-sta6 grown in liquid media under nutrient-replete and nutrient-deprived conditions. Whereby, Fig. 1b shows the results of cell density analysis. Results showed that cells under nutrient deprivations were significantly different from the control (TAP), with TAP-NS being significantly the lowest (p < 0.001) and TAP-N and TAP-S not being significantly different from each other (p > 0.05). In TAP, TAP-N, and TAP-S, the cc5373-sta6 cell density increased after two days, plateaued after four days, and decreased after six days (Fig. 1b). TAP-NS deviated from this trend as its cell density gradually increased throughout the six days (p < 0.05) (Fig. 1b). The biomass was determined by weighing the dry weight of *C. reinhardtii* cells after four days of treatment. The difference in dry biomass among treatments did not differ statistically; and the dry biomass did increase over the course of four days (p < 0.05) (Fig. 1c).

The sustained growth of cc5373-sta6 under all conditions in our experiment could be attributed to continuous light exposure (60–80 µE m^− 2^ s^− 1^). Light is an essential factor in the production of biomass; and half of the microalgae species’ dry weight is carbon and lipid [33]. Results from a time-course analysis of biomass concentration and carbohydrate/lipid contents in *Chlamydomonas* sp. JSC4 revealed significant increases in biomass production and lipid accumulation under continuous light condition compared with that under light/dark cycling condition [34].

Nitrogen and sulfur deprivations are known to cause lipid overaccumulation in *C. reinhardtii* cells [11, 23, 35]. In a study by Yang et al. [16], the combination of 4 g/L sodium acetate supplementation and nitrogen and phosphorous deficiency increased the total fatty acid yield (mg/L) by 93.0% and 150.1% compared to nutrient-depleted and normal culture conditions, respectively. In this regard, it is likely that lipid accumulation might account for the increasing trend in cell density we observed in TAP-NS. Nutrient starvation leads to a slower growth rate and eventually growth arrest, cell division arrest or cell death in microalgae [35, 36]. During nitrogen limitation, cells rearrange their intracellular macromolecular pools, leading to mobilization of carbon to neutral lipid synthesis [36].

Algal cells i.e., cc5373-sta6 with impaired starch metabolism cope differently with oxidative stress than the wild type. For example, under sulfur deprivation conditions, wild-type *C. reinhardtii* produces hydrogen in the light in a sustainable manner via the contribution of two pathways (direct and indirect). In the direct pathway, photosystem II (PSII) supplies electrons to hydrogenase through the photosynthetic electron transport chain, while in the indirect pathway, hydrogen is produced in the absence of PSII through a photosystem I-dependent process. Starch metabolism has been proposed to contribute to both pathways by feeding respiration and maintaining anoxia during the direct pathway and by supplying reductants to the plastoquinone pool during the indirect pathway [37, 38]. Under sulfur deprivation conditions, Chochois et al. [38] found that in cc5373-sta6 PSII-independent hydrogen production was significantly reduced. They also reported that the sta6 mutant uses acetate as substrate to maintain anaerobiosis during the process of hydrogen production by the PSII-dependent pathway. They also observed that induction of hydrogenase activity is severely decreased in sta6 mutant upon dark anaerobic adaptation. On the other hand, no major difference was observed in hydrogen production between the wild type and the starch-deficient mutant sta6 under light anaerobic adaptation [38].

Algal cells subjected to sulfur deprivation were reported to exhibit a less severe response than nitrogen-deprived cells, corresponding to cellular recycling by autophagy and better accumulation of stress marker molecules (carotenoids, TAGs, etc.) [17]. However, our results showed no significant difference in cell density between the –N and –S conditions. In addition, cc5373-sta6 subjected to TAP-NS displayed a gradual increase in cell density during the six-day period. The cc5373-sta6 has been reported to produce significantly higher levels of lipid accumulation than controls under nitrogen deprivation [10, 36]. The increased cellular reactive oxygen species (ROS) play a dual role in signaling lipid biosynthesis and inducing autophagy to recycle specific cellular components [10, 39]. Thus, the increase in ROS caused by the deprivation of N and S could cause a faster induction of lipid production to allow cells to aggressively maintain their survivability.

To achieve optimal value, harvesting processes should not interfere with the quality of biomass by causing cell rupture or loss of cellular contents. In this study, cells subjected to all four treatments accumulated an increase in biomass (dry weight) from day 0 to day 4. Conversely, Salas-Montantes et al. [24] determined that mutant cells deprived of sulfur or of nitrogen had a decrease in biomass compared to the TAP medium control; the biomass was determined in cells overexpressing DNA-binding One Finger (Dof11), a transcription factor that has multiple roles in the regulation of plant physiological processes. Although the biomass decreased over time, the decrease was not significantly different between the sulfur and nitrogen cells [24]. In our study, there was an overall increase among all treatments when comparing Day 0 to Day 4 (p < 0.05). This observation can possibly be explained by the use of the starchless mutant cc5373-sta6 in our study. When microalgae suffer environmental stress, their proliferation slows down, and they begin to produce energy storage products in the form of neutral lipids or starch [40, 41]. The cc5373-sta6 strain does not produce starch [27]; thus, we can assume that the increase in biomass was a result of algal lipid overaccumulation. An increase in the amount of lipids possibly contributed to the observed increase in biomass in our study.

#### Effects on chlorophyll content

**Fig. 2:**
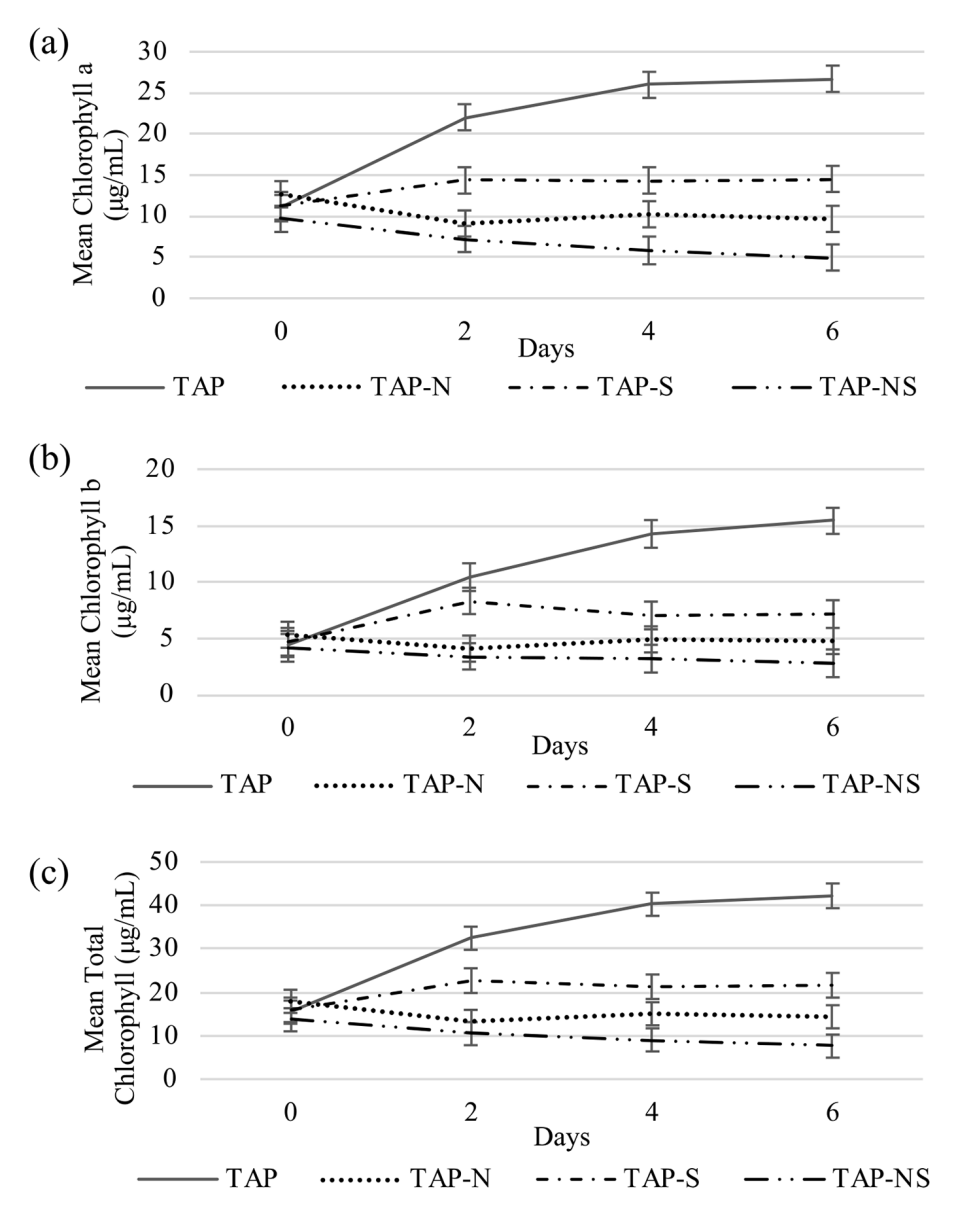
Chlorophyll content analysis of cc5373-sta6 under conditions of nutrient deprivation (-N, -S and –NS); (**a**) chlorophyll a content analysis (**b**) chlorophyll b content analysis (**c**) total chlorophyll content analysis. All analyses were performed every 48 h for six days. Bars in (**a**), (**b**), and (**c**) represent 95% confidence intervals

When subjected to nutrient deprivation, photosynthetic functions are restricted in *C. reinhardtii* cells. In this study, nutrient deprivation conditions significantly lowered chlorophyll a, b and total chlorophyll content compared to the control (p < 0.05) (Fig. 2a-c). The results showed significant differences in chlorophyll a and total chlorophyll among all treatments at day 4 and day 6 (p < 0.05) (Fig. 2a-c). The highest chlorophyll content (a, b, and total chlorophyll) was observed in TAP (control) starting at day 4, whereas –NS was observed to have the significantly lowest amount of chlorophyll a and total chlorophyll content (p < 0.05) starting at day 4. Although cells under nutrient deprivation showed a significantly lower amount of chlorophyll, these cells still maintained their chlorophyll throughout the six days of nutrient deprivation (Figs. 1 and 2). These results indicated that nutrient deprivation likely restricted the cell’s ability to maintain photosynthetic activities, which could result in the alteration of their metabolic pathways to sustain their survivability.

*C. reinhardtii* starchless mutant cells subjected to nutrient deprivation displayed significantly lower chlorophyll content than the control (Fig. 2). In addition, we did not observe a significant difference in chlorophyll content across days; there was no increase in chlorophyll content under all nutrient-deprived conditions compared to the control (Fig. 2). It was reported that nitrogen deprivation in marine microalgae inhibited chlorophyll accumulation as early as 24 h [17, 42]. Nitrogen starvation alters the biosynthesis of cellular pigments, i.e. chlorophylls and carotenoids [16, 34]. During nitrogen deprivation, the synthesis of amino acids is affected, which in turn limits the synthesis of 5-aminolevulinic acid. These changes result in decreased levels of chlorophyll in cells [35]. Cells exposed to nitrogen stress can mobilize chlorophyll molecules and other pigments as an internal source of nitrogen [35, 43]. Recently, researchers have been utilizing melatonin to facilitate the management of cell growth and lipid accumulation in *C. reinhardtii* under nitrogen stress [44]. The use of melatonin weakens nitrogen stress-induced oxidative damage by delaying chlorophyll loss, which is typically observed after 24 h [44]. All of the aforementioned studies support the significantly reduced chlorophyll accumulation we observed after 48 h of nitrogen deprivation, as well as with combined nitrogen and sulfur.

Interestingly, electron microscopy and TAG accumulation experiments showed that an increase in TAG levels coincided with a reduction in the amounts of chloroplast membrane after 12 h of nitrogen deprivation [44]. Reduction of chloroplast membranes is likely one of the contributing factors to the decrease in chlorophyll content in nutrient-deprived cells. Altogether, these results may suggest that lipid accumulation occurs in our nutrient-deprived cells and could be responsible for the observed increase in cell density and biomass in nutrient-deprived cc5373-sta6 in our experiments.

#### Effects on total protein

**Fig. 3:**
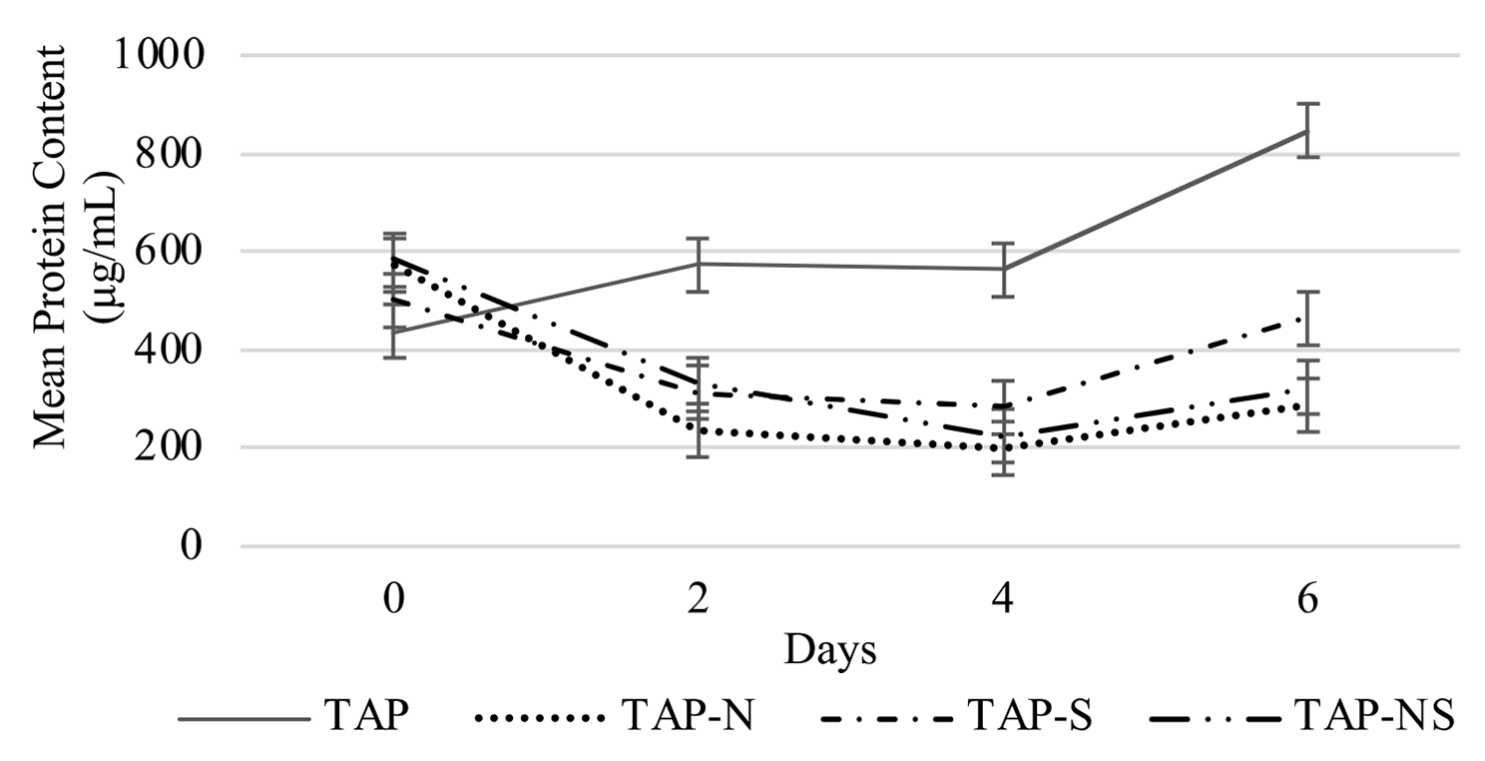
Protein content analysis of cc5373-sta6 under conditions of nutrient deprivation (-N, -S and –NS). Analysis was performed every 48 h for six days. Bars represent 99% confidence intervals

To provide additional evidence that our algal cells were under nutrient-deprived conditions, protein content analysis was performed. Previous studies reported that microalgal cells subjected to either nitrogen or sulfur deficiency have shown an increase in lipid content and a decrease in protein content [41, 45]. A decrease in protein content was also observed in our study. Protein content varied significantly between control and nutrient-deprived cells (p < 0.001). Cells subjected to TAP-NS and TAP-N had significantly the lowest protein content after 6 days (Fig. 3). The results of our protein content analysis indicate that our cells were indeed under nutrient stress.

The increase in TAG accumulation after nitrogen deprivation is known to contribute to a turnover of nitrogen-rich compounds that provide carbon/energy for TAG synthesis [17, 42, 46]. In a study conducted by Sierra et al. [47], a kinetic study was performed to evaluate *C. reinhardtii* protein accumulation under TAP-N and TAP conditions. Protein accumulation decreased after 96 h under nitrogen-deprived conditions. Photosynthetic proteins (i.e., RuBisCo proteins, light harvesting complex proteins) are known to be degraded, decreasing the overall protein content and photosynthetic activity [47]. Additionally, Yu et al. [31] found protein levels to significantly decrease in *C. reinhardtii* subjected to nitrogen deprivation, causing the protein to transform into lipids or carbohydrates. Since cells subjected to nutrient deprivation had decreased chlorophyll and protein contents, we proceeded to analyze lipid body (TAG) accumulation via confocal microscopy analysis.

### Comparison of lipid body number and lipid body size among N-, S- and NS-Deprived cells

**Fig. 4:**
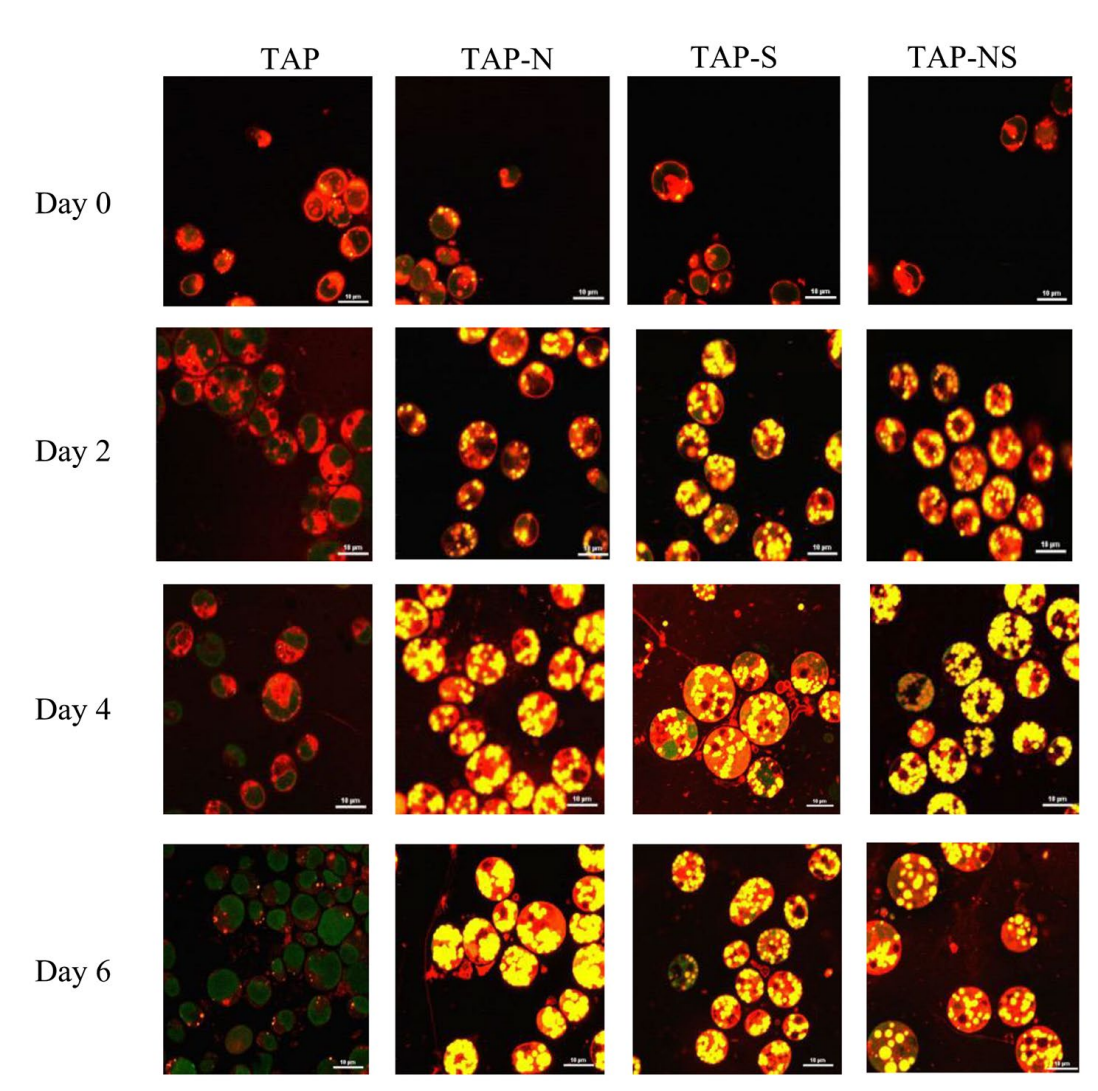
Qualitative lipid body analysis of cc5373-sta6 under nutrient deprivation conditions. Cells were stained with Nile Red and observed under a confocal microscope after 48 h under nutrient starvation treatments (TAP, TAP-N, TAP-S, and TAP-NS) to observe lipid accumulation. Bars represent 99% confidence intervals. Means were determined from three replicates

**Fig. 5:**
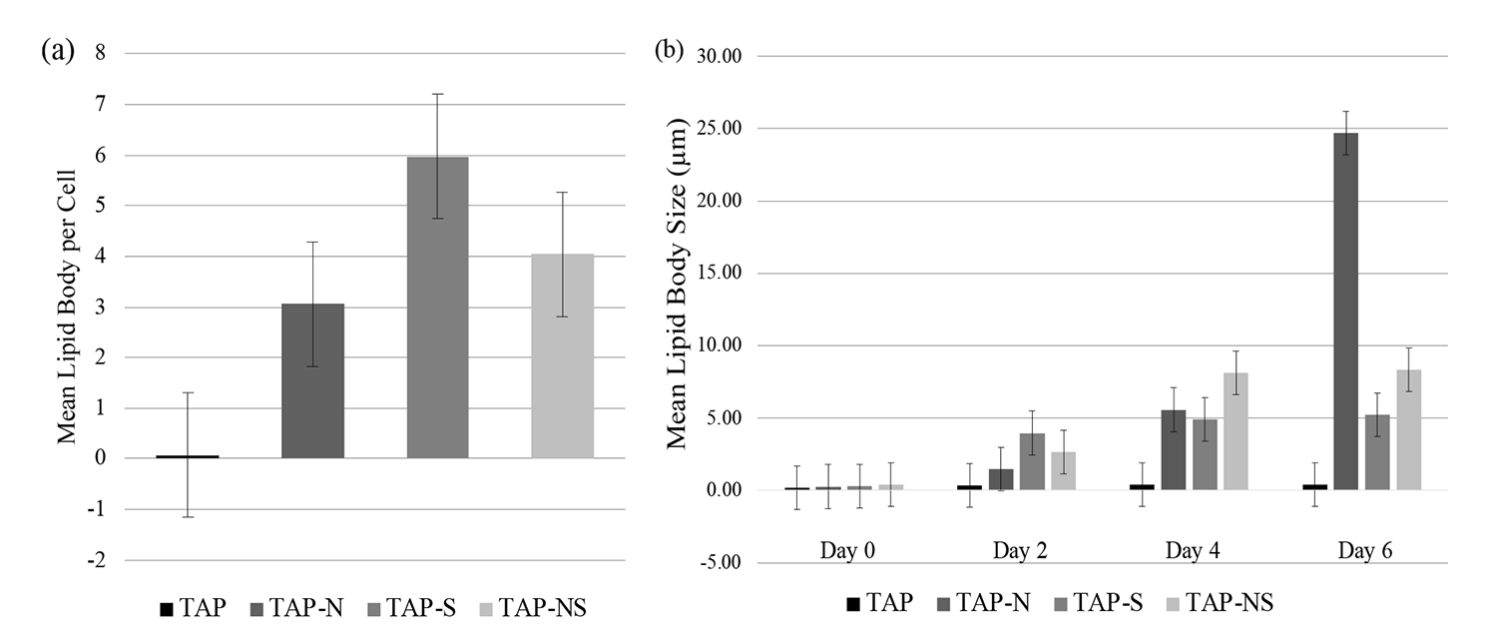
Quantitative lipid body analysis of cc5373-sta6 under nutrient derivation conditions. (**a**) The average lipid body number per cell was determined per frame. The results were pooled by treatments because there was no significant difference among days. (**b**) The average size of lipid bodies per treatment under nutrient deprivation was determined across frames. Bars in (**a**) and (**b**) represent 99% confidence intervals. Means were determined from three replicates

To further characterize the response to nitrogen (N) and sulfur (S) deprivation in the starchless mutant cc5373-sta6, total lipid accumulation was observed via confocal microscopy. Observations were performed every 48 h for six days. Qualitatively, lipid body accumulation representing triacylglycerol (TAG) accumulation was observed after 48 h in all deprivation conditions (Fig. 4). After four days of deprivation, the accumulation of TAGs in TAP-N and TAP-NS was more evident than that on day 2 (Fig. 4). After four days of deprivation, TAP-S cell lipid bodies increased in size and released free-floating lipids (Fig. 4). TAP-S and TAP-NS cells showed the highest amount of lipid bodies, as indicated by the increased yellow staining of lipid bodies (Figs. 4 and 5a). With TAP (control), there was a low yield of TAG accumulation; however, these cells did increase in lipid body number and in the size of lipid bodies over the course of the six-day period (Figs. 4 and 5a and b). After six days of nutrient deprivation, TAP-N cells showed the highest amount of lipid bodies, whereas TAP-S and TAP-NS cells sustained their lipid content (Fig. 4). The deprivation of N, S and the combination of N and S deprivation affected not only the chlorophyll and protein content of the cell but also lipid synthesis.

The number of lipid bodies present in each cell was counted in ten randomized fields of view (1024 × 1024) to obtain an average number of lipid bodies per cell. Lipid bodies (droplets) were also measured using Nikon NIS-Elements confocal software. The size of lipid bodies was measured every 48 h for six days across ten fields of view (1024 × 1024) to obtain an average lipid body size. Over time, the number of lipid bodies per cell did not differ significantly. Although the lipid body number per cell was not significantly different among the nutrient deprivation conditions, they were still significantly different from the TAP control (p < 0.01) (Fig. 5a).

In regard to lipid body size, the trend for average lipid body size across four levels of days varied significantly among treatments (p < 0.01) (Fig. 5b). After four days of nutrient deprivation, there was no significant difference observed among nutrient-deprived cells, and after six days, TAP-N and TAP-NS showed significantly larger lipid bodies than TAP-S and TAP. Interestingly, after six days, TAP-N cells exhibited the largest lipid body size among all treatments, and all treatments were significantly different from each other (Fig. 5b). Cells subjected to both nitrogen (N) and sulfur (S) deprivation displayed its highest lipid body size after four to six days of nutrient deprivation. These results seem to indicate that N deprivation allows for an increase in lipid body number along with a significantly larger size of lipid bodies compared to S deprivation.

Cells subjected to nutrient deprivation accumulate lipids as a result of stress. In this study, the overall accumulation of TAG, as represented by lipid bodies, increased significantly in all nutrient-deprived conditions compared to the control. Studies by Sathe et al. [25] observed that cells grown in the absence of nitrogen accumulate significantly higher amounts of lipids; the results were assessed by Nile red fluorescence intensity. In addition, transmission electron microscopy results demonstrated that under N deprivation, chloroplasts are degraded into smaller sphere-like subcompartments with cytoplasmic lipid droplets being formed [17, 25]. Kamalanathan et al. observed no significant difference among nutrient-deprived cells; cells deprived of S exhibited the highest number of lipid bodies present, and increased cell enlargement was observed [35]. Cells deprived of S after four days exhibited free-floating lipid droplets as a result of cell bursting (Fig. 4). Compared with S-deprived cells, N-deprived cells displayed a lower number of lipid bodies per cell and less cell enlargement. This may correlate with the greater metabolic stress that N-starved cells undergo [17, 35, 46].

Microalgal cell compositions such as lipid content were reported to be influenced as well by light [28]. It was also known that lipid accumulation decreases under light/dark cycling relative to that of continuous illumination [34]. In a study by Kato et al., the cultivation of *Chlamydomonas sp.* JSC4 resulted in higher lipid content under continuous light at optimal light intensity of 300 µmol m^− 2^ s^− 1^ compared to light/dark conditions [34]. In addition, an excess of light illumination to microalgae can induce lipid accumulation rapidly; this is a self-defense system for photo-oxidative damage [48]. In a study that compared the effect of light versus nitrogen deficiency it was shown that exposing a *C. reinhardtii* strain CC-124 (mt2 nit1 nit2) culture to saturating light (SL) of 150 µmol m^− 2^ s^− 1^ under a nonlimiting CO_2_ concentration in turbidostatic photobioreactors induces a sustained accumulation of lipid droplets (LDs) without compromising growth. In addition, the result was a higher lipid productivity than N starvation. The study also showed that the polar membrane lipid fraction of SL-induced LDs contains approximately 70% plastidial lipids, in contrast to N starvation-induced LDs, which contain approximately 60% lipids of endoplasmic reticulum origin [49]. In another study by Zhao et al., the interaction of light intensity and nitrogen concentration on lipid production of *C. reinhardtii* was reported to be significant. This result shows that the interaction of these two factors should be considered at the same time when investigating the accumulation of lipid in *C. reinhardtii* [50]. For future studies, it would be interesting to examine and compare the biomass and lipid yields of *C. reinhardtii* cc5373-sta6 with *C. reinhardtii* cc849 at 100 mg.mL- nitrogen content and 30 µmole m^− 2^ s^− 1^. These are the optimal nitrogen content and light intensity respectively for lipid accumulation in cc849 from Zhao et al.’s study. It would also be interesting to examine optimal sulfur content and light intensity for lipid accumulation in cc5373-sta6.

Fatty acid profiles change with nutrient deprivation [28, 51]. For example, in *C. reinhardtii* cc-400, levels of 18: 3 (9, 12 and 15) and 16: 4 in TAGs increased with N deprivation between 3 and 8 days. Interestingly, 18: 3 (5, 9 and 12) transiently increased with P deprivation but with a decrease in 16: 4 and 18: 3 (9, 12 and 15) [52]. It is also known that if the cells were at nitrogen or phosphorus limited conditions, the relevant enzymes that carry out desaturation and elongation reactions will be produced at a smaller amount, leading to decreased in polyunsaturated fatty acids’ production [52]. In the microalga, *Dunaliella salina*, at iron deprivation, the most abundant fatty acid was linolenic acid (C18:3). In contrast, palmitoleic acid (C16:1) was observed at the highest level during a nitrogen deprivation [53].

The most common fatty acids contained in biodiesel are palmitic, stearic, oleic, linoleic, and linolenic acids [24, 28, 54]. This set of major fatty acids matches with the profiles of fatty acids produced by microalgae [28]. Thus, studies on methods that can enrich the microalgal oil with these beneficial fatty acids is highly important. Based on previous studies mentioned above, we hypothesize that N, S and NS deprivations will have different effects on the fatty acid composition in *C. reinhardtii* cc5373-sta6. Therefore, for future studies, the determination of the fatty acid contents of cc5373-sta6 under N, S and NS deprivations, e.g., fatty acid methyl ester (FAME) analysis by gas chromatography, could determine if cc5373-sta6 will meet the required fatty acid composition for biodiesel production.

### Expression analysis of Acetyl-CoA carboxylase and phosphoenolpyruvate carboxylase

Previous studies of the gene expression levels of enzymes of lipid biosynthesis in *C. reinhardtii* under nutrient deprivation were done to help further understand the potential of microalgae for commercialized biofuel production. These included diacylglycerol acyl transferases (DGATs), acetyl-CoA synthetase (ACS), Acetyl-CoA carboxylase (ACC) and phosphoenolpyruvate carboxylases (PEPC1 and PEPC2 ) [22, 55–57] These enzymes were reported to be affected by N deprivation and have affected lipid metabolism. All these studies were done under N deprivation in the wild-type strain and; with ACC and PEPC as the mostly studied enzymes. Thus, to further establish the effects of nutrient deprivations, -N, -S, and -NS on lipid or TAG accumulation in mutant cc5373-sta6, the expression levels of ACC and PEPC in TAG biosynthesis were investigated in our study.

RNA isolation was performed every 48 h over six days. The yield of RNA from cells subjected to TAP medium after all 48 h intervals was substantial to carry out a genetic analysis; however, cells from our nutrient-deprived treatments had low RNA yields. An analysis of total RNA levels by Park et al. [40] showed a decrease in RNA after 6 h of N starvation; this was attributed to the degradation of purines and pyrimidines from RNA breakdown that appears to contribute N for protein synthesis during the first 24 h of deprivation. Metabolic pathways of *C. reinhardtii* cells were reported to be altered after 4 h of nutrient deprivation [40]. Thus, in our experiments, RNA isolation and determination of gene expression levels were performed at 8 and 24 h intervals.

Schmollinger et al. [46] reported that the highest mRNA abundance of two-protein subunits of acetyl-CoA carboxylase (ACC) were at 4, 8, 12 and 24 h. The 8 h and 24 h time frames are the beginning stages of purine and pyrimidine degradation [40]. Therefore, we found it optimal to isolate RNA at no longer than 24 h before nucleotides were significantly degraded. RNA isolated from cells after 8 and 24 h of nutrient deprivation gave a high yield and purity between 1.7 and 2.0. Previous studies that isolated RNA from *C. reinhardtii* have reported a purity range of 1.7-2.0 [58–60].

**Fig. 6:**
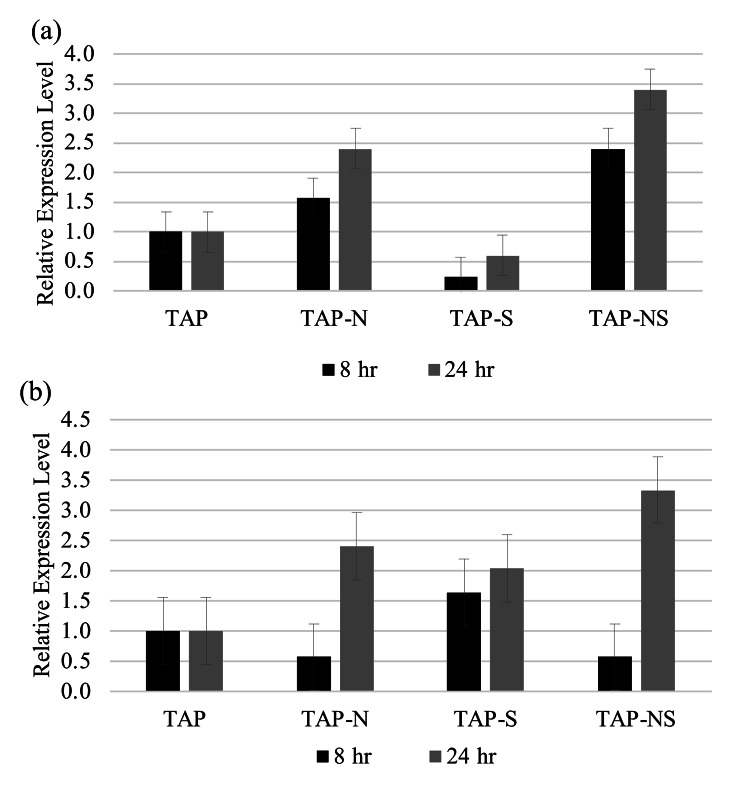
ACC and PEPC expression in cc5373-sta6 under nutrient derivation conditions after 8 and 24 h. (**a**) ACC fold change was determined using the ${2}^{-\varDelta \varDelta \text{C}\text{t}}$ method. (**b**) PEPC fold change was determined using the ${2}^{-\varDelta \varDelta \text{C}\text{t}}$ method. For (**a**) and (**b**), expression levels above 1.000 compared to the TAP control are considered upregulated; expression levels below 1.00 are considered downregulated. Mean values were determined from three replications. Bars represent 99% confidence intervals

RNA isolates were reverse transcribed into cDNA and used for amplification to assess the expression levels of ACC and PEPC. The average Ct value for actin was 18.0. After normalizing our genes of interest to our housekeeping gene, the Ct values of ACC and PEPC in the TAP control exhibited averages of 22.0 and 25.2, respectively. Cells subjected to TAP-NS media had the highest increase in ACC expression level, followed by TAP-N. Both of these treatments were significantly different from the control at both 8 and 24 h (p < 0.01) (Fig. 6a). Conversely, cells subjected solely to S deprivation displayed a decrease in ACC expression after 8 and 24 h that was significantly different from that of the control (p < 0.01). After 8 h of NS deprivation, the expression of ACC increased two-fold compared to that in the TAP control (p < 0.01). Likewise, after 24 h of deprivation, cells subjected to NS deprivation had increased ACC expression, a three-fold increase compared to the TAP control (p < 0.001) (Fig. 6a). At 8 h, TAP-N showed an increase in ACC expression level, although it was not significantly different from the TAP control. However, the 24 h TAP-N group showed an increase in ACC expression compared to the TAP control group after 24 h (p < 0.01) (Fig. 6a).

After 8 h, cells subjected to TAP-N and TAP-NS conditions exhibited a decrease in the expression of PEPC that was not significantly different from the TAP control (Fig. 6b). Conversely, cells subjected to S deprivation expressed an increase in PEPC compared to the TAP control at both the 8 and 24 h time intervals (Fig. 6b). It is interesting to note that after 24 h, both TAP-N and TAP-NS deprivation conditions increased PEPC expression levels; N deprivation increased two-fold (p < 0.01), and NS deprivation increased three-fold (p < 0.01) (Fig. 6b). The highest PEPC expression was in TAP-NS after 24 h, and the lowest expression of PEPC was in TAP-S after 24 h. After 24 h, TAP-NS- and TAP-N-treated cells showed a significant difference in expression compared to the TAP control at 24 h (p < 0.001).

ACC is a key rate-limiting enzyme that catalyzes the first step in the synthesis of fatty acids [22]. It catalyzes conversion of acetyl-CoA and CO_2_ into malonyl-CoA. ACC overexpression in *C. reinhardtii* cw-15 has been shown to directly increase the synthesis of fatty acids [61]. In sta6, Krishnan et al. reported an accumulation of malonyl CoA indicating a reversal of inhibition of ACC relative to the wild-type [62]. It was also observed that ATP accumulation was higher in sta6; this probably inhibits AMP-dependent protein kinase, reducing the level of phosphorylation of ACC, and thus keeps ACC in the active form. In this regard, it was recommended that an overexpression of ACC would need to occur in parallel with the suppression of AMP-dependent protein kinase phosphorylation activity. Therefore, studies on increasing activities of acyl transferases to further improve lipid yields from malonyl CoA would become more relevant in sta6.

The pattern of ACC in fatty acid biosynthesis has been thoroughly investigated and it has been proposed that increased ACC activity is an effective method of stimulating the accumulation of lipids [22, 55]. Based on the results of our gene expression analysis and previous findings from the literature, it would be interesting to determine an optimal N or NS concentration and light intensity for lipid accumulation in cc5373-sta6 and their effects on the activities of ACC. There is no report yet on ACC activity in *C. reinhardtii*, although not done yet in *C. reinhardtii*, ACC enzyme assays were done with other organisms. The most commonly used assay uses a radioactive HCO_3_^−^ assay [63] and another method makes use of a spectrophotometric assay [64–66].

Xu et al. [22] studied the role of the ACC gene under nitrogen deprivation by coculturing *C. reinhardtii* with a nitrogen-fixing aerobic bacterium, *Azobacter chroococcum*. The expression levels of ACC in the co-culture grown under N deprivation were higher than those in pure *C. reinhardtii*. Although these cells were grown with a nitrogen-fixing bacterium, these cells still exhibited an increased amount of lipids as per FAME analysis that coincided with their increased expression of ACC [22]. ACC was reported to be significantly upregulated (1.87 ±0.05-fold) after 6 h of salt (NaCl) stress and then declined slightly after 12 h [67]. These results were similar to the results of our study, whereby there was an increase in ACC expression after 8 and 24 h of N and NS deprivation. The addition of salt and the deprivation of nutrients are both stresses that elicit an increase in lipid biosynthesis [22, 24, 67]. Our ACC gene expression analysis results provide additional evidence that nutrient deprivation, specifically N- and NS-deprivations, can upregulate the expression of ACC, a gene involved in the fatty acid biosynthesis pathway (Fig. 6a).

Phosphoenolpyruvate carboxylase (PEPC) is involved in regulating photosynthesis and respiration, replenishing amino acid metabolism, and catalyzing the formation of oxaloacetate to pyruvate. PEPC catalyses the irreversible β-carboxylation of phosphoenolpyruvate (PEP) to form oxaloacetate. Two PEPC isoforms (PEPC1 and PEPC2) were purified from *C. reinhardtii*. PEPC1 and PEPC2 were reported with respective specific activities (SA) of 22 and 18 µmol min^− 1^ mg^− 1^protein [68]. These activities were similar to the SAs of the recombinant PEPC (CrPpc1, CrPpc2), each of which is transcribed in vivo and encodes a fully active, recombinant PEPC 25 and 22 µmol min^− 1^ mg^− 1^protein. By applying genetic engineering technologies, PEPC have been successfully inhibited to allow its four-carbon substrate to be utilized in the formation of lipid synthesis [69, 70]. Thus, by inhibiting and altering the expression of PEPC, researchers have been able to increase the lipid accumulation rate. In sta6, PEPC becomes more interesting due to a reported fourfold increase in PEP concentration. PEP in sta6 is diverted towards pyruvate formation leading towards acetyl-CoA to to malonyl CoA to synthesize lipids [70].

In this study, the levels of expression of PEPC were determined under N-, S- and NS-deprivation conditions after 8 and 24 h. Our results indicated that after 8 h of deprivation in both nitrogen-deprivation treatments (N and NS), PEPC expression decreased compared to that in the TAP control (Fig. 6b). On the other hand, cells subjected to sulfur deprivation for 8 h displayed an increase in PEPC expression (Fig. 6b). These findings correlate with the delayed response that cells subjected to sulfur deprivation have as a result of autophagy and recycling cellular components and organelles [17, 35]. It was only after 24 h of N- and NS-deprivation that PEPC expression was found to be significantly upregulated (p < 0.01) (Fig. 6b). On the other hand, cells subjected to TAP-S have upregulated PEPC expression levels in both 8- and 24-h intervals compared to the control, TAP; however, this was not significantly different (Fig. 6b).

In the study by Xu et al. [22], the levels of expression of the PEPC gene in N-deprived cells were assessed at days 0, 1, 5 and 9. After one day of deprivation, PEPC expression levels were shown to increase in *C. reinhardtii* cells; however, after 5 and 9 days of N deprivation, PEPC expression levels decreased significantly. Although our transcriptional analysis was conducted over the course of 8 and 24 h, the increase in expression of PEPC was similar to the results observed after one day of N deprivation in the study by Xu et al. [22]. Subsequent days of nutrient deprivation are known to decrease PEPC expression levels, demonstrating that N limitation inhibits the expression of the PEPC gene. Although our study did not report on the effect of nutrient deprivation beyond 24 h, the effect of nutrient deprivation on PEPC expression levels should be mentioned.

To further support the effect of PEPC inhibition on lipid synthesis, a confocal microscopy analysis conducted by Deng et al. [71] demonstrated that knockout of phosphoenolpyruvate carboxylase isoform 1 (CrPEPC1) increased the lipid content by 20–39% after six days of cultivation. The results from this study also demonstrated that the mRNA expression of CrPEPC1 decreased by 74–98%, indicating the effectiveness of their RNAi silencing constructs [71]. Utilizing artificial microRNA technology to inhibit PEPC genes, Wang et al. [72] dramatically increased the total fatty acid content by 29–49% with an increased content of C16-C22 fatty acids. These studies further support that when PEPC is inhibited, pyruvate will be converted to acetyl-CoA via pyruvate dehydrogenase to facilitate lipid synthesis. Thus, nitrogen may act as a signaling molecule to regulate the expression of specific genes when subjected to nutrient stress [22, 71, 72]. With decreased expression of PEPC, lipid accumulation will increase dramatically, especially under nitrogen-deficient conditions.

## Conclusions

The results of this study showed that *C. reinhardtii* cc5373-sta6 subjected to nitrogen and sulfur deprivation accumulated lipids comparable to those of previous studies. Levels of expression of genes that regulate lipid metabolism were shown to correlate to the increase in lipids observed via confocal microscopy. After six days, TAP-N cells exhibited the largest lipid body size among all treatments. In addition, our results seem to indicate that N deprivation allows for an increase in lipid body number along with a significantly larger size of lipid bodies compared to S deprivation. On the other hand, S deprivation allows for a significant increase in lipid body number when compared to N deprivation.

Based on our findings, it would be optimum and thus recommended to cultivate cc5373-sta6 cells in any of the nutrient deprived conditions (-N, -S or –NS) for four days where there is cell growth, and both a high number of lipid bodies and a larger size of lipid bodies are produced. The results of this study will contribute to the understanding of a more feasible microalgae-driven lipid production. It will specifically contribute to the body of knowledge that will potentially allow for the design of an efficient, rapid, and economically viable strategy for enhancing lipid production in *C. reinhardtii*.

## Methods

### Strains, cell culture and harvest

The cc5373-sta6 strain of the *C. reinhardtii* mt^−^ progeny of the original BAFJ5 sta6 starchless mutant crossed into a 21gr background was obtained from the Chlamydomonas Resource Center (www.chlamy.org). Cells were maintained in Tris-acetate-phosphate (TAP) media agar plates at 25°C under continuous low intensity white light (60–80 µE m^− 2^ s^− 1^). Cells were grown in 50 mL TAP medium, which included acetate (17.4 mM) as a carbon source and Tris-base (20 mM) as a buffer, under the same conditions as cell maintenance on a rotary shaker at 120 rpm and used as the algal cell stock for N, S and NS deprivation treatments. A calibration curve was constructed by measuring the optical density (OD) at 750 nm (Bausch and Lomb, Model Spectronic 20) over the course of six days, and OD750 = 0.8 was used for log phase [30, 32].

Once the cells reached the log phase, the cells were harvested via centrifugation at 2,900 g for 5 min at 10^o^C (FIBERLite TM F15-8 × 50c). The supernatant was removed, and pelleted cells were washed thrice with their respective media: TAP-N media to remove nitrogen, TAP-S media to remove sulfur and TAP-NS media to remove nitrogen and sulfur [22]. The pelleted cells were resuspended in 50% of their original volume using the respective media and centrifuged at 2,900 g for 5 min (FIBERLite TM F15-8 × 50c) to harvest the cells. After all the washing steps, the pellet was resuspended in 100 mL of its respective media, and 25 mL of cells was transferred into 250 mL sterilized flasks and placed on a rotary shaker. Cells were collected from these flasks for analysis of growth, chlorophyll, protein, lipid bodies and gene expression. All analyses were conducted in duplicate for each of three blocks.

### Cell growth determination

#### Cell density measurement

Cell density measurement was used to determine and compare cell growth among treatments. Two 1 mL samples were collected from each flask and transferred into a cuvette. The cuvette was placed in a spectrophotometer (Bausch and Lomb, Model Spectronic 20), and the OD was measured at 750 nm [30, 32]. Sterile TAP, TAP-N, TAP-S, or TAP-NS media was used to blank and calibrate the spectrophotometer for each treatment. Cell density measurements were recorded every 48 h across a six-day period.

#### Biomass determination

Fifty milliliters of cells were taken for day 0 of biomass collection. The cells were centrifuged at 1,600 g for 10 min (Beckman Coulter Microcentrifuge 20 B30139), and the supernatant was discarded. The pelleted cells were washed with Millipore water and centrifuged. Pelleted cells were resuspended in 5 mL of Millipore water and transferred to empty preweighed 15 mL centrifuge tubes. The cells were centrifuged, and the supernatant was removed. The cells were dried in an oven at 80 °C for 48 h and weighed. This protocol was repeated at day 4 of starvation [22].

### Chlorophyll content determination

For chlorophyll a, b and total chlorophyll determination, two 3 mL cell cultures were collected from each flask and transferred into sterile 15 mL conical centrifuge tubes. Samples were centrifuged at 1,600 g for 10 min (Beckman Coulter Microcentrifuge 20 B30139), and the supernatant was discarded. (1) The pelleted cells were added to 3 mL of 80% acetone (v/v). (2) Each tube was vortexed at the max setting for 30 s to resuspend the cells completely. (3) The cells were centrifuged at 1,600 g for 10 min (Beckman Coulter Microcentrifuge 20 B30139), and the supernatant was collected. Steps (1) to (3) were repeated until the pellet turned white and the supernatants at each extraction were combined. These steps allowed for chlorophyll extraction.

One milliliter of the chlorophyll-containing supernatant was transferred into a cuvette, and the OD at 750 nm was measured for total chlorophyll, and the OD at 663.6 nm and OD at 646.6 nm were measured for chlorophyll A and chlorophyll B content, respectively [73]. Samples were collected every 48 h across a six-day period.

### Protein content determination

Protein content was measured using a Thermo Scientific Pierce BCA protein assay kit. Algal cells were harvested and centrifuged at 7,500 g for 10 min (Beckman Coulter Microcentrifuge 20 B30139). The supernatants were discarded. One milliliter of 15 mM KH_2_PO_4_ (pH 4.5) and 2 mL of 20% NaOH were added to each tube and shaken for 30 s. The tubes were placed in boiling water for 10 min followed by centrifugation at 7,500 g for 10 min. The supernatants were collected and used for the assay. Bovine serum was used as the standard protein. All optical densities (OD) were measured using a Tecan Microplate Reader at 560 nm.

### Lipid body analysis

#### Qualitative analysis

Two 3 mL-cell cultures from each treatment were placed in a 15 mL-centrifuge tube, followed by centrifugation at 2000 g for 5 min, and the supernatant was removed. One microliter Nile Red (Sigma-Aldrich) stock solution containing 1 mg/mL acetone was added to the pelleted cells and incubated for 5 min at room temperature [72]. An 8 µL aliquot was placed on a clean glass slide, covered with a coverslip and placed in an incubator at 37°C for 10 min. The samples were observed with a Nikon ECLIPSE Ti2 Series confocal microscope using a laser excitation line at 488 nm, and an emission was collected between 620 and 700 nm; chlorophyll fluorescence was captured with a laser excitation line at 633 nm, and an emission was collected between 620 and 700 nm [74]. Images were merged and pseudocolored using Nikon NIS-Elements software [72]. Scans of the algal cells were taken with a 60x oil immersion objective at a pixel resolution of 1024 × 1024 in an 8-bit format (pixel intensity range 0-255). The laser transmission and scan settings remained constant in all scans [74].

#### Quantitative analysis

The number of algal cells with and without lipid bodies was counted along with the total number of lipid bodies present per ten fields of view [75, 76]. An average lipid body per cell was determined for every 1024 × 1024 8-bit frame. The average size of lipid bodies per frame was also measured by Nikon confocal software per field of view to compare the treated algal cells with the control [73, 77]. The Nikon NIS-Elements confocal software counted and summed all the pixels in the chosen fields that were above the selected threshold of brightness, thereby computing the total area of the above-threshold entities [72]. The threshold was adjusted by highlighting lipid bodies; if they varied in brightness, the least bright lipid body in the selected field served as the threshold baseline [72]. The output of each calculation yielded the average lipid body area per frame. It should be noted that measurement of lipid body area underestimates the spherical volume and hence will also underestimate the yield. It will be a comparison of lipid body areas that allows for accurate assessment of the relative yields from different samples [72].

### RNA extraction and real-time quantitative PCR

One hundred milliliters of cells was collected at 8 and 24 h intervals. RNA was extracted using TRIzol Reagent (Invitrogen). The concentration and purity of the extracted RNA were measured using a Tecan Microplate Reader at 260 nm and obtaining the ratio of OD at 260 nm:280 nm, respectively. Two micrograms of DNAse-treated and purified total RNA was used for cDNA synthesis using the Superscript^™^ III First-Strand Synthesis System (Invitrogen). Transcriptional levels of acetyl-CoA carboxylase (ACC), phosphoenolpyruvate carboxylase (PEPC), and actin were detected using real-time (RT) quantitative PCR. Primers used for RT-PCR were designed by Xu et al. [22]. RT-PCR was performed using the iTaq Universal SYBR Green Super Mix (BioRad). The actin gene from *C. reinhardtii* was used as an internal control to normalize the differences between the loading amounts of the template. Each PCR contained 1 µL (8 ng) of cDNA, 10 µL of SYBR Green 2x Master Mix, and 1 µL of each gene-specific primer pair (10 mM) to a final volume of 20 µL. PCR was performed as follows: 95 °C for 10 min followed by 40 cycles at 95 °C for 10 s, 60 °C for 1 min, and 72 °C for 30 s. PCR products were analyzed using the Dissociation Curves Software of the CVX96 Touch Real-Time PCR Detection System (Bio-Rad). The ${2}^{-\varDelta \varDelta \text{C}\text{t}}$ method was used to calculate the fold changes of the expressed genes.

### Statistical analysis

For cell growth, chlorophyll content, protein content and lipid body analyses, an analysis of variance associated with a 4 × 4 factorial experiment of randomized complete block design was performed. The factorial arrangement was the result of the four levels of treatment (TAP, TAP-N, TAP-S, and TAP-NS) and four levels of time (Day 0, Day 2, Day 4, and Day 6). For the genetic analysis, an analysis of variance associated with a 4 × 2 factorial experiment of randomized complete block design was performed. The factorial arrangement was the result of the four levels of treatment (TAP, TAP-N, TAP-S, and TAP-NS) and two levels of time (8 and 24 h). A blocking design was implemented to reduce the variability within blocks from external factors and produce a better estimate of treatment effects. To compare the significant main and interaction effects for all experiments in this study, a post hoc test in the form of Tukey’s test and Bonferroni correction was performed using PROC GLM of the SAS 9.4 statistical and SPSS 27 software. The mean results of all studies were generated from three replications, and each replication had at least three trials. The usual levels of type-1 error rates were used (i.e., * if p < 0.05, ** if p < 0.01 and *** if p < 0.001).

## Data Availability

The datasets generated during and/or analyzed during the current study are available from the corresponding author on reasonable request.

## List of Abbreviations

TAP-N: TAP media minus nitrogen
TAP-S: TAP media minus sulfur
TAP-NS: TAP media minus nitrogen and sulfur
